# Silver Nanoparticles (AgNPs) Incorporation into Polymethyl Methacrylate (PMMA) for Dental Appliance Fabrication: A Systematic Review and Meta-Analysis of Mechanical Properties

**DOI:** 10.3390/ijms252312645

**Published:** 2024-11-25

**Authors:** Kacper Galant, Natalia Turosz, Kamila Chęcińska, Maciej Chęciński, Katarzyna Cholewa-Kowalska, Sławomir Karwan, Dariusz Chlubek, Maciej Sikora

**Affiliations:** 1 Faculty of Medicine, Medical University of Lodz, Al. Kościuszki 4, 90-419 Łódź, Poland; kacpergalant.ld@gmail.com; 2 National Medical Institute of the Ministry of Interior and Administration, Wołoska 137 Str., 02-507 Warsaw, Poland; natalia.turosz@gmail.com (N.T.); maciej@checinscy.pl (M.C.); sikora-maciej@wp.pl (M.S.); 3 Department of Maxillofacial Surgery, Hospital of the Ministry of Interior, Wojska Polskiego 51, 25-375 Kielce, Poland; 4 Department of Glass Technology and Amorphous Coatings, Faculty of Materials Science and Ceramics, AGH University of Science and Technology, Mickiewicza 30, 30-059 Kraków, Poland; checinska@agh.edu.pl (K.C.); cholewa@agh.edu.pl (K.C.-K.); 5 Faculty of Applied Sciences, WSB Academy, Cieplaka 1C, 41-300 Dąbrowa Górnicza, Poland; 6 Institute of Applied Sciences, WSB Merito University in Poznan, Sportowa 29, 41-506 Chorzow, Poland; 7 Department of Oral Surgery, Preventive Medicine Center, Komorowskiego 12, 30-106 Kraków, Poland; 8 Department of Maxillofacial Surgery, Regional Specialized Children’s Hospital, 10-561 Olsztyn, Poland; slawek.karwan@gmail.com; 9 Department of Biochemistry and Medical Chemistry, Pomeranian Medical University, Powstańców Wielkopolskich 72, 70-111 Szczecin, Poland

**Keywords:** polymethyl methacrylate, silver, nanoparticles, dentures, mechanical tests

## Abstract

Polymethyl methacrylate (PMMA), widely used in orthodontics and dentures, exhibits suboptimal mechanical properties, including flexural strength (FS), impact strength (IS), and tensile strength (TS), which are critical for ensuring durability, resistance to fracture, and overall functionality of dental appliances. Silver nanoparticles (AgNPs) are a promising filler due to their antimicrobial properties. This pre-registered PRISMA-compliant meta-analysis compared the mechanical properties of PMMA/AgNP composites to pure PMMA, considering mean particle size and concentration. Of 360 records from ACM, BASE, PubMed, and Scopus, 35 studies were included (κ = 0.91), covering 88, 38, and 11 tests on FS, IS, and TS, respectively. FS increased only between 30–70 nm (*r* = 0.00; *ρ* = 0.03; *R*^2^_deg2_ = 0.13). IS remained higher for <80 nm and increased between 15 and 25 nm (*r* = −0.41; *ρ* = −0.28; *R*^2^_deg2_ = 0.59). TS favored 55 nm but had limited data (*r* = −0.24; *ρ* = 0.63; *R*^2^_deg2_ = 0.99). FS decreased with increasing wt%, showing no discernible trend (*r* = −0.22; *ρ* = −0.34). IS increased within 0.0–4.0 wt%, particularly 0.5–2.0 wt% (*r* = −0.15; *ρ* = −0.35; *R*^2^_deg2_ = 0.54). TS peaked at 0.5–2.0 wt% (*r* = −0.20; *ρ* = −0.24; *R*^2^_deg2_ = 0.91). Optimal mechanical properties of PMMA/AgNP likely fall within 15–70 nm mean nanoparticle size and 0.5–4.0 wt% concentration.

## 1. Introduction

Polymethyl methacrylate (PMMA), commonly referred to as acrylic, is extensively utilized in various fields, including engineering, healthcare, and dentistry [1]. It is recognized as one of the most frequently chosen dental materials [2]. It is most commonly used for producing complete and partial denture bases. Nevertheless, it is also employed in the manufacture of artificial teeth, impression trays, temporary crowns, obturators for cleft palates, occlusal splints, printed or milled casts, treatment planning matrices, denture relines, and repairs [1].

The main component of the removable orthodontic appliance is the base plate, typically made of self-curing acrylic resin [3]. The base plate connects all the parts, including clasps and other metal components [3,4,5]. Its primary function is to apply forces to the teeth and bone structures [4,5]. Because removable appliances are meant to be worn daily for at least a dozen hours, they need to be durable and dimensionally stable [3]. PMMA is also used to make relaxation splints. These devices must withstand the significant chewing forces associated with bruxism, which can reach up to 720 N during maximum clenching force [6]. This is why materials science needs to continue developing new modifications of PMMA to enhance this material’s strength. Acrylic dentures have many advantages including a low material cost, good aesthetics, and ease of use. However, they are susceptible to fracture due to their poor mechanical properties [7].

Research is being conducted worldwide to add various other components such as reinforcement fibers: carbon, aramid, nylon, polyethylene, polypropylene, and glass, to improve the poor properties of acrylic [1]. Various nanoparticles, ceramics, and metals such as Ag, TiO_2_, ZrO_2_, and Al_2_O_3_ are also used [1,8]. Moreover, new methods for making and processing PMMA are being introduced. Attempts have been made to produce PMMA prostheses using CAD/CAM technology, improving their properties. Studies confirm positive results in such features as hardness, flexural strength, modulus of elasticity, and impact strength [9,10,11,12]. In addition, durability and mechanical properties have been improved compared to heat-cured PMMA [9,13].

Acrylic devices used in dentistry, as a result of use by the patient, are colonized by opportunistic bacteria present in the oral cavity, which leads to the development of dental infections [14]. The silver nanoparticles (AgNPs) inhibit the growth of bacteria such as *S. aureus*, *S. sobrinus*, *L. casei*, *P. aeruginosa*, *A. baumannii*, and *P. mirabilis* [15,16]. In addition to antibacterial, antifungal AgNP properties against *C. Albicans* have been demonstrated. Pathogen adhesion and biofilm formation limiting is an important stage in stopping the pathogenesis of stomatitis in patients using dentures [17,18,19,20,21,22].

This study aims to evaluate the impact of AgNPs on the mechanical properties such as flexural, impact, and tensile strength of PMMA composites, concerning the nanoparticle size and concentration, focusing on dental applications. We pose the following null hypothesis “The addition of AgNPs does not change the mechanical properties of PMMA”.

## 2. Materials and Methods

This systematic review was developed under the PRISMA 2020 guidelines. The protocol was prospectively published in the Open Science Framework Registries (osf.io/yqftx, accessed on 16 October 2024) [23].

### 2.1. Eligibility Criteria

The systematic review included reports containing the results of durability and antifungal tests of PMMA incorporated with AgNPs, compared with analogous tests of PMMA without nanoadditives. Detailed eligibility criteria are given in Table 1.

### 2.2. Sources and Search Strategy

Medical databases were searched on 24 August 2024, using the following search engines: (1) ACM, (2) Bielefeld Academic Search Engine (BASE), (3) PubMed, and (4) Scopus (Table A1) [24,25,26,27]. The search strategy was developed by two (K.G. and M.C.) and endorsed by the remaining authors. It was formulated based on the material’s name, the names of various dental products made from this material, synonyms for incorporating AgNPs, and the specific mechanical forces examined in the studies. Finally, the following query was used in all search engines: “(pmma OR polymethyl OR methacrylate OR acrylic) AND (denture OR dentures OR prosthesis OR prostheses OR tray OR trays OR appliance OR appliances OR retainer OR retainers OR crown OR crowns OR guard OR guards OR splint OR splints) AND (ag OR agnp OR nag OR silver OR silvery OR argent OR argentine) AND (nano OR nanoparticle OR nanoparticles) AND strength AND (flexural OR tensile OR impact OR transverse)”.

### 2.3. Selection Process

Identified records were manually deduplicated by one of the authors (K.G.), who consulted with another author (M.C.) in case of doubt. Two authors (K.G. and K.C.) performed blinded screening based on titles and abstracts. Cohen’s *κ* coefficient was calculated using MedCalc (Version 23.0.2; MedCalc Software Ltd., Ostend, Belgium) to express inter-rater agreement. Records consistently qualified or assessed equivocally were promoted to the next stage. Two authors (K.G. and N.T.) performed a full-text evaluation, finally qualifying or disqualifying reports based on the above-defined eligibility criteria. Another author (M.C.) had the final vote in decision discrepancies. Papers rejected at this stage were cited in this systematic review and, in case of exclusion, the reasons were provided. The authors used a Rayyan tool (Version 2024.08.29; Rayyan Systems Inc., Cambridge, MA, USA) to enhance, but not automatize, the deduplication and selection processes.

### 2.4. Data Collection Process and Data Items

One author (K.G.) performed manual data extraction without using automation tools. Two other authors (N.T. and M.C.) jointly verified the collected data. The authors (K.G., N.T., and M.C.), divided into roles similarly, transferred the collected numerical data into spreadsheet using Google Workspace software (Version 2024.08.23; Google LLC, Mountain View, CA, USA). Disputes were resolved by consensus, with the casting vote of one author (M.C.). Table 2 summarizes the extracted data and their corresponding definitions outlined in this review. Whenever multiple time-dependent testing variants were present, the data closest to 30-day conditioning was extracted. For trend visualization purposes, the mean nanofiller particle size was estimated based on the reported extreme values in studies that did not provide this variable.

### 2.5. Effect Measures and Synthesis Methods

All outcomes were presented relatively due to the different measurement methods used by the individual research teams. The measure of effect for the strength variables was the percentage ratio between the result of the material with AgNP and the result of pure PMMA. For all variables, the values for unenriched PMMA were assumed to be 100%. The data were collected in tables and presented on charts, providing trendlines when possible. Google Sheets (Google LLC, Mountain View, CA, USA) and Microsoft Excel (Microsoft Corporation, Redmond, WA, USA) programs were used to analyze and visualize the results. Pearson’s *r* and Spearman’s *ρ* correlation coefficient values were calculated. The significance level adopted was *α* = 0.05. Linear, quadratic, and logarithmic regression models were tested.

### 2.6. Selection Results

**Figure 1:**
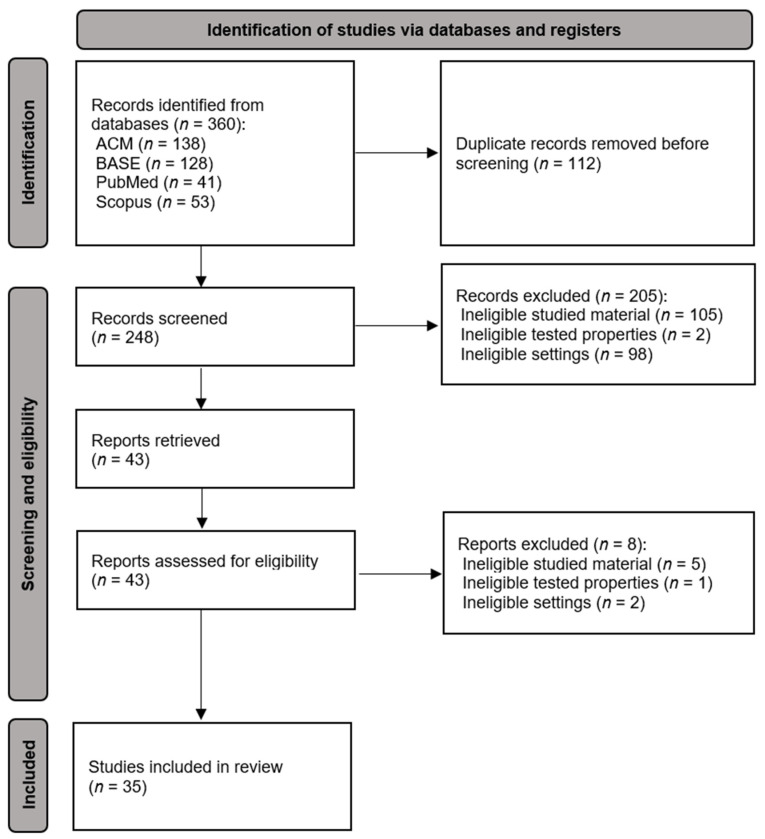
Flow diagram.

The selection process is presented in Figure 1. Of the 360 records identified, 112 duplicates were rejected. Screening of titles and abstracts resulted in the exclusion of 205 records with the compliance of the two judges being *κ* = 0.91. Out of 43 reports assessed for eligibility in full text, eight were ineligible due to studied material, tested properties, or settings. The rejected reports at the full-text assessment stage are presented in Table A2 with the reasons for exclusion.

## 3. Results and Discussion

### 3.1. Results of Individual Studies

Ultimately, 35 studies were included (Table 3) [17,18,20,21,22,28,29,30,31,32,33,34,35,36,37,38,39,40,41,42,43,44,45,46,47,48,49,50,51,52,53,54,55,56,57]. The flexural strength was reported in 26 studies, while the impact and tensile strength were assessed in 11 and 7 studies, respectively. The specimens had varying dimensions, ranging from 2 to 200 mm. In some samples, the incorporated AgNP particles were smaller than 10 nm, while in others they were as large as 100 nm. Fabrication methods differed between the studies, with the predomination of heat-curing.

### 3.2. Results of Syntheses

#### 3.2.1. Correlation Analyses

No strong (>0.50) linear correlations are observed between the strength values and the mean particle size or nanofiller concentration (Table 4). The observed weak linear correlations are negative. A statistically significant positive correlation was noted between the average AgNP particle size and tensile strength in the rank approach. Moreover, statistically significant negative rank correlations were noted between the additive concentration and the other two types of strength.

#### 3.2.2. Regression Analysis

**Figure 2:**
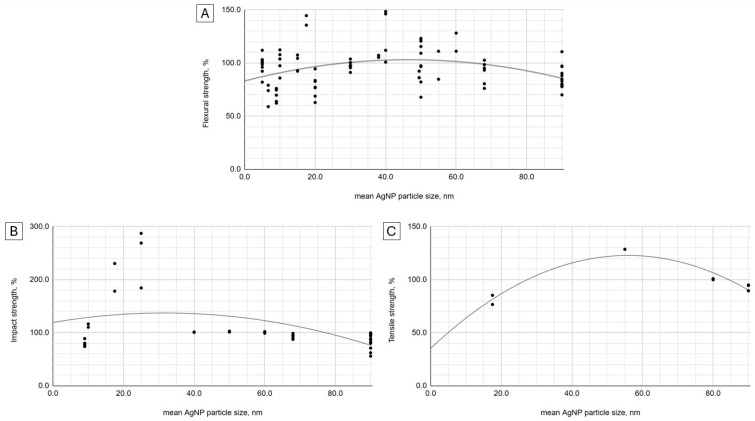
Flexural (**A**), impact (**B**), and tensile (**C**) strength to AgNP mean particle size.

The average AgNP particle size showed a weak quadratic relationship (*R*^2^ = 0.13) with the flexural strength, suggesting an improvement over the baseline within the 30–70 nm range (Figure 2). A stronger (*R*^2^ = 0.59) relationship of the same kind was observed for impact strength values, favoring particles below 80 nm. A polynomial trend line for the relationship between tensile strength and average AgNP particle size was strongly marked (*R*^2^ = 0.99). The latter analysis suggested an improvement in the material tensile strength provided that the average nanoadditive particle size was included in the range of 30 to 85 nm.

**Figure 3:**
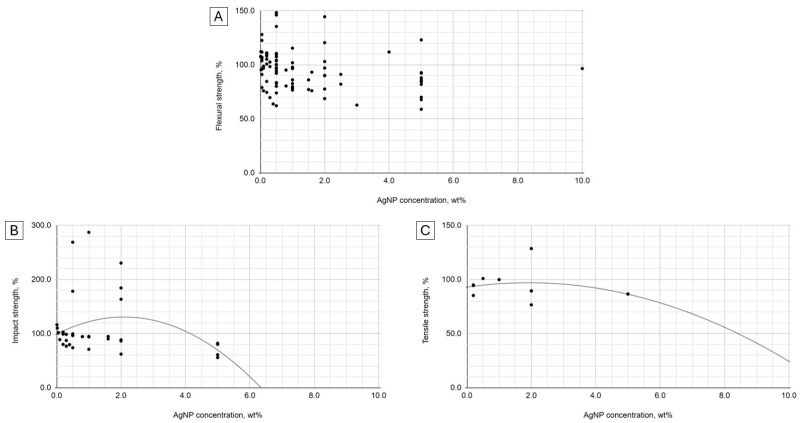
Flexural (**A**), impact (**B**), and tensile (**C**) strength to AgNP concentration.

The relationship between flexural strength and AgNP concentration did not fit the regression models tested (Figure 3). The impact strength data were somewhat quadratically related (*R*^2^ = 0.54) to AgNP concentration. The peak impact strength for 2 wt% concentration was noted. A much stronger (*R*^2^ = 0.91) trend of the same type was observed for tensile strength, favoring 2 wt% AgNP.

In the theoretical model, the peak strength values and optimal AgNP particle sizes are expected to be 103% at 47 nm, 137% at 32 nm, and 123% at 56 nm for flexural, impact, and tensile strength, respectively. The calculated hypothetical maximum strength values and optimal nanoparticle concentrations are 131% at 2.1 wt%, 97% at 1.9 wt% for impact and tensile strength, respectively.

### 3.3. Interpretation

Flexural strength is the ability to resist deformation under an applied load [58]. Various factors can lead to denture fracture; therefore, the material requires a high flexural strength [59]. To achieve this, attempts have been made to add various particles and materials to the PMMA, such as Au, Pt, copper oxide nanoparticles, glass fibers, butadiene styrene, or nanodiamonds [32,60,61,62]. In the present systematic review, the best results were obtained by Kavyashree et al. incorporating 0.5% AgNPs [52]. Similar results were also obtained by Anas et al., where adding 0.5% or 2% of AgNPs increased the discussed parameter by approximately 35% and 44%, respectively [44]. On the other hand, some studies showed different results. Francisco Nunes de Souza Neto et al. noted a significant decrease in flexural strength when incorporating 5% AgNPs [42]. This concentration also caused a similar effect in the study by Alla et al. [34]. In many studies, flexural strength decreases with increasing concentration of AgNPs [7,33,34,46]. However, in the study by Nam et al., this parameter increases with a higher percentage of AgNPs [32].

Despite numerous examples of more or less marked changes in flexural strength due to the enrichment of PMMA with AgNP, no trend is observed in the general comparison with the concentration of the nanofiller. Pearson’s correlation with the concentration of nanoparticles is weak and negative, and Spearman’s is on the border between statistical significance and negative [63]. There is no correlation of this strength variable with the size of nanoparticles, but a weak quadratic relationship indicates a subtle improvement in strength in the 30–70 nm range. In summary, the entire meta-analysis conducted for flexural strength suggests no relationship or decrease with the increase in the additive concentration and possibly better outcomes for middle-sized nanoparticles.

Apart from satisfactory flexural strength, dentures should also have high impact strength to prevent breakage if accidentally dropped [64]. Mithran et al. observed a significant increase in the impact strength of modified PMMA with AgNP. The best results were obtained when acrylic resin was modified with 1 wt% AgNP [49]. Significantly higher concentrations resulted in lower impact strength values. Alla et al. obtained different results. Regardless of the AgNP concentration, the impact strength decreased, albeit again mainly at the highest concentration, based on the results of one experimental study only [53]. In addition, in the study by Srivastava, 5% AgNP caused a significant decrease in this variable. Higher concentrations also caused discolorations of denture bases [53]. Palaskar et al. used small concentrations of AgNP (below 0.5%) incorporated into PMMA. The results showed that the impact and flexural strength decrease as the wt% of AgNPs increases. However, the best efficacy against *C. albicans* was observed in the group with the highest concentration of AgNPs, which aligns with two other studies [21,65,66].

All correlations indicate that the impact strength decreases with an increasing AgNP concentration and particle size. However, these are weak relationships with the strongest on the border of statistical significance. Fairly evident (R^2^ > 0.5) quadratic trends indicate the peak impact strength for particles below the 4th size quantile and concentrations close to 2 wt%.

Tensile strength was the least frequently assessed variable in analyzed studies. The best results were achieved by Aljafery et al., where incorporating 2% AgNP into PMMA increased tensile strength by almost 30% [38]. According to a study by Ziąbka et al., adding these particles did not affect the tensile strength [47]. The largest decrease in tensile strength occurred in the study by Ghaffari et al., where PMMA was reinforced with 2% AgNPs [29]. Incorporating 5% of AgNP caused a smaller decrease in this variable [31].

The correlation between tensile strength and the concentration of AgNPs was weak. However, some research suggests that the effect of AgNPs depends on their concentration [29]. The results of the meta-analysis were on the one hand supported by the most modest data set, but on the other hand, showed the strongest trends. The quadratic fitting curves again indicated 2 wt% and the middle two quantiles of particle size as directions for finding a material with optimal strength properties.

### 3.4. Generalizability

The size and concentration of silver nanoparticles (AgNPs) influence the mechanical properties of the PMMA matrix. Nanoparticles within the size range of 15–70 nm are considered to demonstrate improved dispersion within the polymer matrix, potentially resulting in a more homogeneous distribution and reduced agglomeration. This uniformity in dispersion may facilitate enhanced stress transfer between the matrix and the nanoparticles, thereby contributing to improved flexural and tensile strength [17,18,20,21,22,28,29,30,31,32,33,34,35,36,37,38,39,40,41,42,43,44,45,46,47,48,49,50,51,52,53,54,55,56,57].

Optimal concentrations of AgNPs, estimated between 0.5–4.0 wt%, are thought to provide sufficient interfacial bonding without promoting particle clustering, which could act as stress concentrators and compromise the structural integrity of the composite. Conversely, excessive nanoparticle loading may disrupt the polymerization process, potentially leading to voids or defects undermining the composite’s mechanical properties [17,18,20,21,22,28,29,30,31,32,33,34,35,36,37,38,39,40,41,42,43,44,45,46,47,48,49,50,51,52,53,54,55,56,57].

The nonlinear relationships observed in this study, including the absence of a consistent trend in flexural strength with increasing AgNP concentration and the variability in impact strength, can be linked to the above mechanisms. Particle agglomeration at higher AgNP concentrations likely acts as a stress concentrator, reducing the stress transfer efficiency within the PMMA matrix. Additionally, the interfacial bonding between AgNPs and the PMMA matrix, particularly pronounced for smaller nanoparticle sizes, has been identified as a critical factor in enhancing the mechanical properties of the composite [17,18,20,21,22,28,29,30,31,32,33,34,35,36,37,38,39,40,41,42,43,44,45,46,47,48,49,50,51,52,53,54,55,56,57].

The interplay between nanoparticle distribution, interfacial bonding dynamics, and agglomeration behavior provides a plausible explanation for the observed nonlinearity. These factors, previously analyzed, highlight the complexity of optimizing AgNP parameters to achieve consistent improvements in mechanical performance [17,18,20,21,22,28,29,30,31,32,33,34,35,36,37,38,39,40,41,42,43,44,45,46,47,48,49,50,51,52,53,54,55,56,57].

In summary, adding AgNPs is intended to provide antimicrobial activity to the composite and, within the indicated ranges, does not impair its mechanical properties. Adhering to the proposed size and concentration ranges may maintain the durability and fracture resistance of the PMMA matrix, making it a promising material for dental applications. Nonetheless, further research is required to elucidate the precise mechanisms underlying these effects.

### 3.5. Limitations, Strengths, and Applicability

The presented systematic review only included research reported in English. Therefore, some valuable publications may have been overlooked. This meta-analysis considered only three strength tests. Many included studies did not assess impact and tensile strength values. Additionally, there were differences between the fabrication methods of acrylic resin.

The available data on individual strength properties are limited by the scarce number of primary studies and the methodologies employed. The sample fabrication methods varied, but conventional heat-curing was dominant, which constitutes the applicability of the results in clinical applications. This is particularly true for the production of dental prostheses, where this method is widespread. The possibilities of alternative fabrication are less well represented in primary studies on the AgNPs incorporation into the PMMA structure and therefore require further experimental studies.

The included studies utilized varying testing protocols, complicating the results’ comparability and integration into a unified meta-analysis. The lack of standardization in tensile strength testing methods led to inconsistencies in reported values, which may affect the interpretation of results and conclusions. This limitation underscores the need for further research using unified and standardized procedures, such as those recommended by international standards (e.g., ISO or ASTM). A repetitive approach would allow for a more precise determination of the influence of AgNP size and concentration on mechanical properties, including the least represented tensile strength, thereby improving the reliability of future meta-analyses.

Another limitation of this meta-analysis is the high heterogeneity of observation durations across the included studies, which prevents the incorporation of this variable into the analysis. Future research should prioritize standardizing observation periods and consider the time-dependent effects of material performance. Additionally, experimental studies should aim to replicate oral cavity conditions as closely as possible to improve the clinical relevance and applicability of the findings.

Despite these limitations, our findings provide valuable insights into potential trends and mechanisms by which silver nanoparticles affect the mechanical properties of PMMA. The paper addresses the current issue of unsatisfactory mechanical properties of PMMA. It discusses how incorporating antimicrobial AgNPs into acrylic resin can influence its mechanical properties [67]. The review analyzed various parameters and attempted to find correlations and trends concerning the concentration and particle size of AgNPs. The searches with board-scoped engines included numerous scientific databases, guaranteeing systematic review comprehensiveness and enhancing meta-analysis reliability. Its important strength lies in the high inter-reviewer agreement (κ = 0.91), which reflects the robustness and reliability of the study selection and evaluation processes. This strong concordance highlights the rigor of our methodological approach and bolsters confidence in the validity of the findings.

These findings significantly imply PMMA-based dental materials development, particularly in addressing the balance between mechanical performance and antimicrobial functionality. One of the primary motivations for incorporating AgNPs into PMMA is their antifungal and antibacterial potential, which is highly desirable in dental applications to reduce the risk of microbial colonization and associated infections. This meta-analysis provides critical insights into whether such additions can be implemented without compromising the material’s mechanical properties. Our results indicate that with optimized AgNP size and concentration, PMMA composites can maintain or even enhance fracture resistance. This demonstrates the feasibility of developing PMMA composites that integrate antimicrobial benefits while retaining their structural integrity.

To further enhance the clinical applicability of these findings, future research should focus on standardizing methodologies for incorporating AgNPs into PMMA and evaluating the long-term biocompatibility and safety of these materials. Such efforts will be essential for translating the insights from this study into practical guidelines for the production and use of PMMA composites in dental practice.

## 4. Conclusions

Based on the findings from the identified 35 studies with 119 sample sets, an average particle size in the range of 15–70 nm and a concentration of 0.5–4.0 wt% appear to be the most suitable parameters for maintaining or enhancing the flexural, impact, and tensile strength of PMMA/AgNP composites. To ensure the optimal mechanical strength of acrylic composites, further studies on antimicrobial activity should concentrate on the specified particle sizes and silver nanoparticle concentration ranges. These studies should consider the peak theoretical values of 47 nm (wt% not specified), 32 nm and 2.1 wt%, and 56 nm and 1.9 wt% to ensure optimal flexural, impact, and tensile strength, respectively.

## Figures and Tables

**Table 1:** Eligibility criteria.

	Inclusion Criteria	Exclusion Criteria
Studied material	PMMA modified with AgNPs	Other nanoparticle additions, e.g., vanadium
Reference material	PMMA without AgNP additive	Other nanoparticles added
Tested properties	Tensile, flexural, or impact strength	Non-quantitative tests
Settings	Primary in vitro studies	Non-English reports, preprints, conference papers

**Table 2:** Data items and their definitions.

Data Item	Description
Specimen	Shape and size of the block of material being used
Fabrication method	Type of material being used and its preparation method
AgNP size particle	Range and mean diameter of silver nanoparticles
AgNP concentration	Concentration (wt%) of AgNPs
Flexural strength	Maximum stress value at which the material can withstand bending without destruction
Tensile strength	Maximum stress value at which the material can withstand stretching or pulling without being destroyed
Impact strength	Amount of energy that a material can absorb and its resistance to stress at high speed

**Table 3:** Results of individual studies.

First Author, Publication Year	Specimen	Fabrication Method	AgNP Size, nm (Range, Mean)	AgNP Concentration, wt%	Flexural Strength, % *	Impact Strength, % *	Tensile Strength, % *
Sodagar, A., 2012 [28]	Block 50 × 10 × 3.3 mm	Auto polymerizing acrylic resins	N/S, 38	0.05 0.20	107.22 105.34	N/S	N/S
Ghaffari, T., 2014 [29]	Block 2 × 20 × 200 mm	Heat-curing acrylic resin	<35, N/S	0.20 2.00	N/S	N/S	85.33 76.68
Bajracharya, S., 2014 [22]	Block 64 × 10 × 3.3 mm	Heat-curing acrylic resin	<99, N/S	0.50 1.00 1.50	92.37 86.21 86.20	N/S	N/S
Hamedi-Rad, F., 2014 [30]	Block 2 × 20 × 200 mm—Contradictory data	Heat-curing acrylic resin	N/S, N/S	5.00	N/S	N/S	86.83
Ghaffari, T., 2015 [31]	Block 2 × 20 × 200 mm	Heat-curing acrylic resin	N/S, N/S	5.00	N/S	N/S	86.58
Nam, K.Y., 2016 [32]	Block 25 × 2 × 2 mm	Heat-curing acrylic resin	<10, N/S	0.10 0.50 1.00 2.00 4.00	98.87 100.34 102.04 103.17 112.00	N/S	N/S
Köroğlu, A., 2016 [33]	Block 65 × 10 × 2.5 mm and 50 × 6 × 4 mm	Heat-curing acrylic resin	N/S, 68	0.30 0.80 1.60	98.48 95.27 93.33	87.50 94.48 90.42	N/S
	Block 65 × 10 × 2.5 mm and 50 × 6 × 4 mm	Microwave-polymerizing acrylic resin	N/S, 68	0.30 0.80 1.60	102.69 80.55 76.16	98.81 94.69 94.88	N/S
Alla, R.K., 2017 [34]	Block 65 × 10 × 3 mm	Heat-curing acrylic resin	80–100, N/S	0.50 1.00 2.00 5.00	110.63 78.66 90.34 84.88	N/S	N/S
	Block 65 × 10 × 3 mm	Heat-curing acrylic resin	80–100, N/S	0.50 1.00 2.00 5.00	80.29 79.81 77.76 70.08	N/S	N/S
	Block 65 × 10 × 3 mm	Heat-curing acrylic resin	80–100, N/S	0.50 1.00 2.00 5.00	83.06 96.70 97.16 88.11	N/S	N/S
Vijay, A., 2018 [35]	Block 65 × 10 × 3	Heat-curing acrylic resin	N/S, 30	0.50	96.83	N/S	N/S
	Block 65 × 10 × 3	Heat-curing acrylic resin	N/S, 30	0.50	103.81	N/S	N/S
	Block 65 × 10 × 3	Heat-curing acrylic resin	N/S, 30	0.50	100.43	N/S	N/S
Turki, M.M., 2018 [36]	Block 80 mm × 10 mm × 4 mm	Heat-curing acrylic resin	<20, N/S	0.01 0.02	107.86 112.35	116.57 110.50	N/S
Siripanth, J., 2018 [37]	Block 64 mm × 10 mm × 3.3 mm	Heat-curing acrylic resin	20–40, N/S	0.025 † 0.05 † 0.075 † 0.1 †	95.59 91.18 97.06 97.06	N/S	N/S
Aljafery, A.M.A., 2018 [38]	Block 20 cm × 2 cm × 0.2 cm	Heat-curing acrylic resin	N/S, 55	2.00	N/S	N/S	128.65
Oyar, P., 2018 [39]	Block 60 × 7 × 4 mm	Heat-curing acrylic resin	N/S, 40	0.05 † 0.20 †	111.97 100.85	101.48 100.74	N/S
	Block 60 × 7 × 4 mm	Heat-curing acrylic resin	N/S, 50	0.05 † 0.20 †	122.69 109.24	101.48 102.96	N/S
	Block 60 × 7 × 4 mm	Heat-curing acrylic resin	N/S, 60	0.05 † 0.20 †	128.21 111.11	102.22 99.26	N/S
Munikamaiah, R.L., 2018 [40]	Block 65 × 10 × 3 mm	Heat-curing acrylic resin—short curing cycle	10–20, N/S	0.50 5.00	107.56 92.83	N/S	N/S
	Block 65 × 10 × 3 mm	Heat-curing acrylic resin—long curing cycle	10–20, N/S	0.50 5.00	104.40 92.34	N/S	N/S
Gopalakrishnan, S., 2019 [41]	N/S	Heat-curing acrylic resin	<100, N/S	1.00 2.00 5.00 10.00	115.58 120.63 123.20 96.68	N/S	N/S
Souza Neto, F.N.D., 2019 [42]	Block 60 × 10 × 3 mm	Heat-curing acrylic resin	N/S, 5.8–7.6 ‡	0.05 † 0.50 † 5.00 †	79.14 74.10 58.99	N/S	N/S
Aziz, H.K., 2019 [43]	Block 80 × 9 × 3 mm	Heat-curing acrylic resin—water bath	N/S, 90	0.20 2.00	N/S	N/S	94.51 89.53
	Block 80 × 9 × 3 mm	Heat-curing acrylic resin—autoclave	N/S, 90	0.20 2.00	N/S	N/S	95.04 89.60
Alsukhayri A.A., 2019 [44]	Block 65 × 10 × 2.5 mm—FS 60 × 7 × 4 mm—IS	Heat-curing acrylic resin	<35, N/S	0.50 2.00	135.66 144.63	178.44 230.54	N/S
Alhawiatan, A.S., 2020 [45]	Block 75 × 10 × 2 mm	Light-curing acrylic resin	N/S, N/S	2.00	N/S	163.62	N/S
Takamiya, A.S., 2021 [18]	Block 64 × 10 × 3.3 mm	Heat-curing acrylic resin	N/S, 5	0.05 † 0.50 † 5.00 †	96.15 92.31 82.05	N/S	N/S
	Block 64 × 10 × 3.3 mm	Heat-curing acrylic resin	N/S, 10	0.05 † 0.50 † 5.00 †	103.85 97.44 85.90	N/S	N/S
Pinheiro, M.C.R., 2021 [46]	Block 65 × 10 × 2.5 mm	Heat-curing acrylic resin	N/S, 50	1.00 2.50 5.00	97.43 82.21 67.85	N/S	N/S
Ziąbka, M., 2021 [47]	N/S	Heat-curing acrylic resin	N/S, 80	0.50 1.00	N/S	N/S	101.00 99.98
Gad, M.M., 2022 [17]	Block 65 × 10 × 2.5 mm	Heat-curing acrylic resin	5–60, 20	0.50 1.00 1.50	94.55 82.78 77.20	N/S	N/S
Kaur, L., 2022 [48]	Block 65 × 10 × 2.5 mm	Heat-curing acrylic resin	N/S, N/S	2.50	91.30	N/S	N/S
Vaiyshnavi, W., 2022 [20]	Block 65 × 40 × 5 mm	High-impact injection molded PMMA	N/S, N/S	0.05 0.20	105.83 108.33	N/S	N/S
Mithran, A., 2023 [49]	Block 50 × 6 × 4 mm	Heat-curing acrylic resin	20–30, N/S	0.50 1.00 2.00	N/S	269.01 287.32 184.51	N/S
Srivastava, R., 2023 [50]	Block 60 × 7 × 4 mm	High-impact acrylic resin	N/S, N/S	5.00	N/S	61.16	N/S
Pai, E., 2023 [51]	Block 65 × 10 × 3 mm	Heat-curing acrylic resin	N/S, 20	0.50 1.00 2.00 3.00	83.61 76.78 68.85 62.86	N/S	N/S
Kavyashree, D.K., 2023 [52]	Block 70 × 10 × 3 mm	Heat-curing acrylic resin—short curing cycle	30–50, N/S	0.50	146.25	N/S	N/S
	Block 70 × 10 × 3 mm	Heat-curing acrylic resin—long curing cycle	30–50, N/S	0.50	148.50	N/S	N/S
Alla, K.R., 2023 [53]	Block 50 × 6 × 4 mm	Heat-curing acrylic resin	80–100, N/S	0.50 1.00 2.00 5.00	N/S	99.78 94.17 86.61 80.35	N/S
	Block 50 × 6 × 4 mm	Heat-curing acrylic resin	80–100, N/S	0.50 1.00 2.00 5.00	N/S	96.58 71.22 62.23 55.94	N/S
	Block 50 × 6 × 4 mm	Heat-curing acrylic resin	80–100, N/S	0.50 1.00 2.00 5.00	N/S	97.95 95.0 88.64 82.27	N/S
El-Hussein, I.G., 2024 [54]	Block 65 × 10 × 2.5 mm	Heat-curing acrylic resin	N/S, N/S	2.00 5.00	90.06 84.51	N/S	N/S
Choure R., 2024 [55]	Block 65 × 10 × 2.5 mm	Heat-curing acrylic resin	10–20/30–50 ‡, N/S	1.00	97.93	N/S	N/S
Palaskar, J.N., 2024 [21]	Block 65 × 10 × 2.5 mm 65 × 10 × 3 mm	Heat-curing acrylic resin	6–10, 9	0.10 0.20 0.30 0.40 0.50	76.11 74.65 69.82 63.92 62.28	89.06 80.29 76.98 79.86 74.10	N/S
Emam AN., 2024 [56]	Block 65 × 10 × 2.5 mm	Heat-curing acrylic resin	N/S, N/S	0.50	109.47	N/S	N/S
Sukumaran K., 2024 [57]	Block 64 × 10 × 3.3 mm	Heat-curing acrylic resin—conventional curing	10–100, N/S	0.20	84.75	N/S	N/S
	Block 64 × 10 × 3.3 mm	Heat-curing acrylic resin—autoclave method	10–100, N/S	0.20	111.10	N/S	N/S

N/S—not specified; *—relative to the reference material; †—approximate value; ‡—determined by various methods.

**Table 4:** Pearson’s *r* and Spearman’s *ρ* correlations.

Tested Property Change	Mean AgNPs Size	AgNPs Concentration
Flexural strength—*r*	0.00	−0.22
Impact strength—*r*	−0.41	−0.15
Tensile strength—*r*	−0.24	−0.20
Flexural strength—*ρ*	0.03	−0.34 *
Impact strength—*ρ*	−0.28	−0.35 *
Tensile strength—*ρ*	0.63 *	−0.24

*—strong *r* or statistically significant *ρ.*

## Data Availability

All collected data are included in the content of this article.

## Appendix A

**Table A1:** Search engine scopes.

Engine	Scope, Records
ACM	3,746,876
BASE	406,042,796
PubMed	over 37,000,000
Scopus	over 94,000,000

**Table A2:** Reports excluded at the full-text assessment stage.

First Author, Publication Year	Identifier	Reason for Exclusion
Sodagar, A, 2012 [28]	10.1016/j.jpor.2011.06.002	Ineligible studied material (repair resin)
Li, M., 2022 [68]	10.3390/biomimetics7030138	Ineligible studied material
Kul, E., 2016 [69]	10.1016/j.prosdent.2016.03.006	Ineligible studied material (ineligible particle size)
Altaie, S.F., 2023 [70]	10.17219/dmp/137611	Ineligible tested properties
Nam, K.Y., 2008 [71]	kumel.medlib.dsmc.or.kr/handle/2015.oak/39707	Ineligible settings (non-English report)
Barbur, I., 2023 [72]	10.3390/biomedicines11071965	Ineligible studied material (graphene-Ag additive)
Strzelecka, K., 2023 [73]	10.5114/ps/177085	Ineligible settings (non-English report)
Kaul, S., 2023 [74]	10.7759/cureus.48260	Ineligible studied material
